# Institutional and policy networks in disaster management in the Horn of Africa: insights from Kenya^1^

**DOI:** 10.1111/disa.70031

**Published:** 2025-12-02

**Authors:** Tahira Mohamed

**Affiliations:** ^1^ International Livestock Research Institute Nairobi Kenya

**Keywords:** community disaster committee, coordination, disaster risk management, Horn of Africa, institutional analysis, policy network analysis

## Abstract

Disasters in the Horn of Africa have become more frequent and protracted due to climate change and the compounding factors that restrict resource access, leading to severe food insecurity and livelihood losses. While governmental, international, and regional partners have made significant progress in adopting disaster management policies and frameworks, challenges remain. Limited empirical attempts have been made to understand how diverse actors with differing goals and competing interests interact with one another and influence disaster management and development outcomes. Drawing on qualitative data collected over two years, this paper explores the diverse practices within and across disaster management in the region, using Kenya as a case study, and focusing in particular on drought as a national disaster. It investigates how multiple actors, networks, and other hidden dynamics shape disaster response outcomes. It establishes that various institutional barriers, rigid structures and mindsets, along with shifting priorities and insufficient resources, often result in fragmented drought responses, disjointed coordination, and siloed operations across multiple layers. This study highlights the importance of paying attention to these dynamics and recognising them as key grounds for improvement. It advocates for more introspection and new approaches to collaboration rather than new policy frameworks.

## 1 INTRODUCTION

Globally, an estimated 1.65 and 1.43 billion people were adversely affected by floods and droughts, respectively, between 2000 and 2019 (Browder et al., 2021, p. 17). While critical adaptation and disaster management research has often emphasised coordination between stakeholders as being key for effective disaster preparedness, response, and recovery, disaster response in the Horn of Africa has tended to be sluggish (Levine, Crosskey, and Abdinoor, 2011; Farr et al., 2022), with stakeholder activities often poorly aligned (Mohamed et al., 2025). Multiple disaster management institutions and policies have emerged in the region, including at the intergovernmental, national, county, and village levels. Most of these policies advocate for effective disaster response coordination and resilience building, emphasising pre‐ and post‐disaster recovery (IGAD, 2019; Republic of Kenya, 2025). Drought has been declared a national disaster in the Horn of Africa on various occasions in recent years, notably in 2011, 2017, and 2021 (Hassan, 2017; ADRA et al., 2021). Although this paper uses drought and disaster interchangeably, they are different: drought is a slow‐onset event, whereas disaster can be a sporadic event.

Drought management is anchored on three pillars: (i) effective monitoring and early warnings; (ii) preparedness and mitigation; and (iii) response and recovery plans (UNISDR, 2012; Pischke and Stefanski, 2016). Operationalising these pillars requires strong institutional backing, effective coordination mechanisms, collaboration among actors, and the political will for resource allocation (Pischke and Stefanski, 2016; FAO, 2019). Despite the broadly agreed‐upon theoretical significance of integrated disaster management, limited empirical attempts have been made to understand how diverse actors with differing goals and competing interests operate in relation to each other and influence disaster management and development outcomes (Lee and Park, 2008; Lobo, 2008; Guthiga and Newsham, 2011; Keating and Hanger‐Kopp, 2020; Walker et al., 2024). In undertaking this kind of analysis—with a focus on Kenya and using examples from a recent drought—this paper grapples with the question of how different institutional policies and actors interact with and influence overall disaster management and coordination. It investigates the extent to which policy actors effectively align their approaches to disaster preparedness, response, and recovery.

The differing institutional interests and priorities of agencies in disaster risk management (DRM) can either create barriers or opportunities for policy implementation. A key concept in understanding how this takes place is the notion of ‘brokers and translators’, as put forward by Lewis and Mosse (2006). Such brokers and translators interpret policy objectives and turn them into practical programmes or ‘read the meaning of policies in different institutional languages’ (Mosse, 2005, p. 9). This translation occurs at multiple levels: in the donor or field office, among practitioners, within government agencies, or at the beneficiary community level. In many respects, such individuals are comparable to Roe's (2020) concept of ‘high reliability professionals’. Drawing on critical infrastructure such as electricity and energy, Roe (2020) argues that highly skilled individuals (professionals) ensure a stable flow of resources and avoid the complete collapse of the system they operate. That is, they generate reliability within wider systems through flexible relations and the capacity to mobilise knowledge, resources, and other professionals. What I mean by this is that when the complex systemic challenges involved in disaster response and recovery are effectively addressed, this tends to occur through dynamic collaborations between policy brokers, translators, beneficiaries, and policy enablers, who, in this context, make the system stable and reliable during a crisis by capitalising on their dynamic skillsets.

Drawing insights from the broader disaster response literature, and particularly approaches focused on policy and institutional network analysis (Mosse, 2005; Lee and Park, 2008; Kim and Hossain, 2013), this paper analyses how different actors in the drought management cycle, bound by a standard framework or policy, form networks, coordinate activities, and influence disaster management. Using northern Kenya's Marsabit and Isiolo Counties as a case study, it seeks to unravel some of the ways in which drought management is shaped by interactions between actors at different levels (international, national, county, and community). It assesses the role of coordination among these actors (policy enablers, policy translators/implementors, intermediary committees, and beneficiaries) in enhancing effective disaster response and recovery, especially in the context of global aid cuts. Its overarching objective is to reveal the institutional dynamics that guide disaster response, including who coordinates activities and what key relationships exist among actors whose work is centred on averting disasters, particularly drought. It emphasises the need to capitalise on ‘bonding’ relationships that foster trust and accountability.

The paper begins by outlining its approach, which concentrates on policy networks and institutional analysis. It then assesses Kenya's disaster risk policies and institutions at the national level, evaluating relationships and interactions among actors engaged in policy implementation, and discusses the constraints, tensions, and prospects associated with mainstreaming DRM in both national and local planning. It concludes by synthesising key lessons for policymakers and practitioners to improve disjointed and reactive responses to complex disasters in the Horn of Africa.

## 2 POLICY NETWORK AND INSTITUTIONAL ANALYSIS

Policy network analysis has been widely employed as a means of critically examining disaster management, with a particular focus on large‐scale, sudden, and catastrophic crises, such as hurricanes and earthquakes (Chua, Kaynak, and Foo, 2007; Lee and Park, 2008; Kim and Hossain, 2013; Kapucu and Garayev, 2016). Nevertheless, it remains an underutilised analytical lens in the Horn of Africa, where some of the most widely experienced disasters are predictable and have slow onsets, such as droughts (Keating and Hanger‐Kopp, 2020; Campbell, 2021). Policy network approaches assess relationships and interactions between actors engaged in policymaking processes (Lee and Park, 2008). Indeed, Lee and Park (2008) describe a range of different observable relationships, some characterised by more frequent interaction and meetings and others predisposed to conflict and limited trust, creating hurdles for effective disaster management. These latter kinds of relationship are comparable to those described by von Lubitz, Beakley, and Patricelli (2008), who note that poor information flow within and among actors involved in disaster response is the key disabling feature. They advocate for a ‘network‐enabled capability’ approach, emphasising real‐time, unrestricted information exchange among all participants.

Disaster management takes shape amidst varied processes and dynamics; sometimes, these include a disconnect between government disaster response plans and actual operations (Choi and Brower, 2006). Network analysis is a useful methodological tool for understanding how and why this is the case and unearthing practical insights to help determine how different kinds of relationships shape the deployment of resources (Guthiga and Newsham, 2011). In utilising it, this study categorised actors into three groups: (i) policy enablers (institutions that authorise policy formulation and resource allocation, including government and donors (Lobo, 2008, p. 26); (ii) policy translators (organisations and individuals who interpret policies and turn them into desired interventions, consisting of government departments, development non‐governmental organisations (NGOs), humanitarian agencies, and civil society organisations (CSOs); and (iii) policy beneficiaries (community groups that directly or indirectly benefit from interventions). The paper explores how these actors interact and coordinate their activities for effective drought management, centred on preparedness, coordinated response, and recovery.

Building on this analysis, the paper also investigates how ideological differences, competing objectives, and interests serve to engender control‐oriented technocratic strategies, with NGOs, donors, and practitioners struggling to collaborate amidst often fraught power relations and complex operating environments (cf. Cooley and Ron, 2002; Alsop, 2005; Watkins and Swidler, 2013). In this sense, it aligns with work that has analysed how policies fail or succeed, often depending on the effects of multi‐layered brokerage, ‘hidden’ goals, and system ambiguity (Mosse and Lewis, 2005; Barresi, 2018). This frequently results in critical disconnects between the disaster management policies and the institutions and actors responsible for translating and implementing them.

Such disconnects can be overcome by drawing on broader social capital ideas defined as ‘features of social life—networks, norms and trust—that enable the participants to act together more effectively to pursue shared objectives’ (Putnam, 1995, pp. 664–665). What this means is that the actors within a given homogeneous agency tend to capitalise on mutually binding strong ‘bonding relationships’ (Andrew and Carr, 2013), which are characterised by ‘thick trust’. Yet, ‘bridging relationships’ are often characterised by ‘thin trust’ between heterogeneous groups, enabling participants to ‘get ahead’ in achieving the desired goals. Here, such relationships enhance coordination and information flow to manage disasters more efficiently, with shared responsibility and a collective outcome. While noting the potential downsides of exclusion and inclusion (Maxwell et al., 2016), bonding and bridging relationships can offer a wealth of hope, especially if policy enablers, implementors, and policy beneficiaries see themselves operating in this realm. This requires a radical shift and strong leadership by existing policy institutions and actors active in this space, as discussed to some extent in the ensuing section.

**FIGURE 1:**
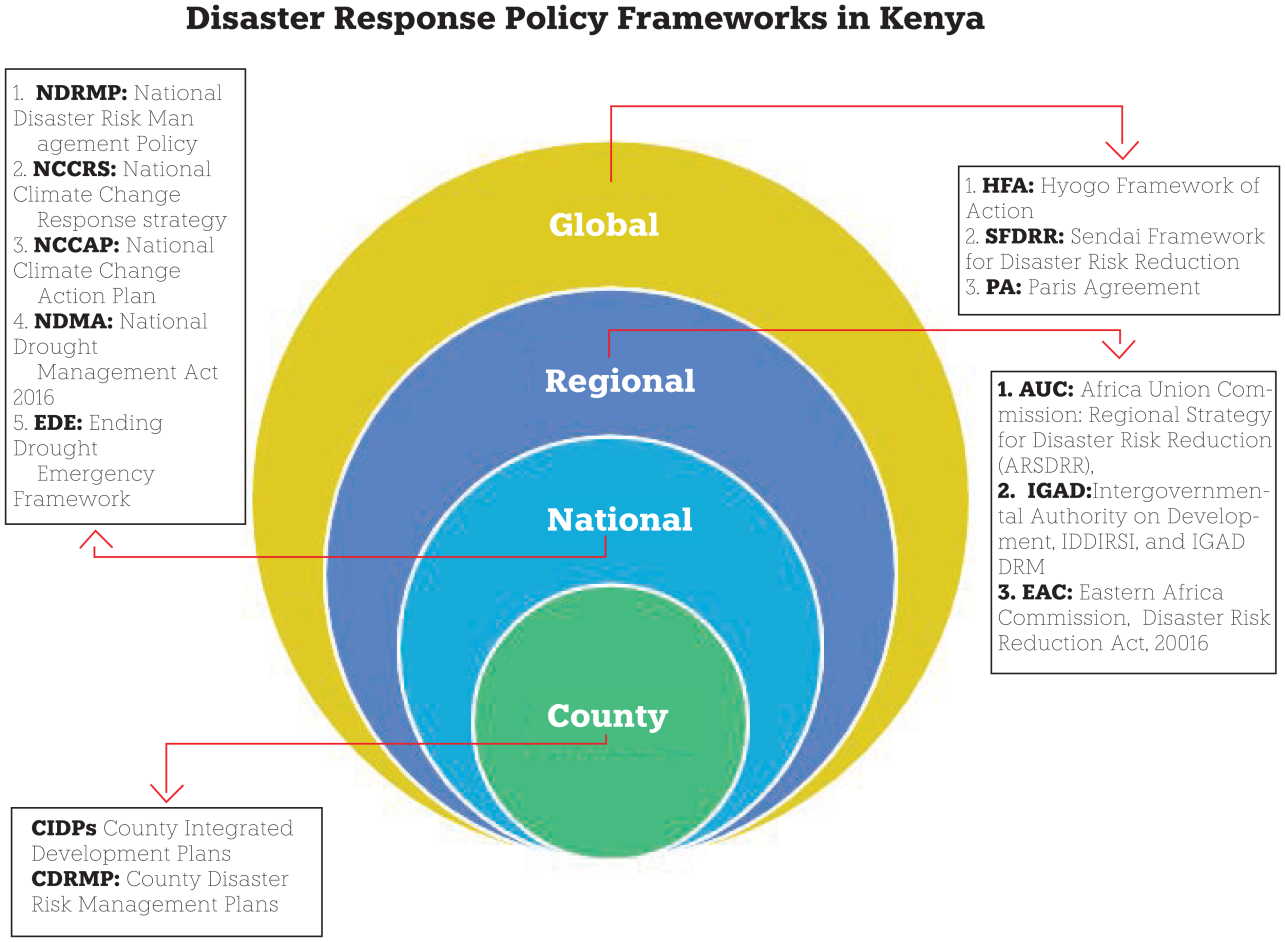
Diagram summarising disaster response policy frameworks in Kenya. **Source:** author.

Effective disaster management also requires a capacity to learn from experience and adapt strategies based on what works. In a broad sense, such a capacity is perhaps limited throughout much of the aid sector, where, as Mosse (2011) points out in *Adventures in Aidland*, idealised objectives are often disconnected from the realities of everyday practice. The kind of critical reflection by both approaches and agendas that might be conducive to progressive internal change is challenging to find across the disaster management sphere in Kenya. Thus, while policies themselves have evolved over the past few decades (see Kenya's ‘progressive’ policy framework, as shown in Figure 1), responses seem passive and disconnected from local practices and knowledge (Derbyshire et al., 2024). This means that the hidden aspirations, competing needs, and conceptualisations of disasters, including drought, are only discernible through careful analysis of relationships between the policy actor's practices and their institutional mandates, often rooted in policy frameworks and strategies.

Kenya's disaster operation systems are spread across multiple agencies, policies, and institutions with responsibilities and mandates that are frequently ambiguous, resulting in overlapping roles. In terms of policy frameworks, Kenya has committed to global and regional frameworks for disaster risk reduction (DRR), including the Hyogo Framework for Action 2005–15, the Sendai Framework for Disaster Risk Reduction 2015–30, and the Paris Agreement. As Kenya's disasters are mostly hydrometeorological and climate‐related, disaster policies align with climate change adaptation (CCA). DRR and CCA share the objectives of reducing vulnerability and enhancing community resilience, reaffirming the goals outlined in the Paris Agreement and the Sendai Framework (UNISDR, 2009). Kenya has also adopted the Africa Regional Strategy for Disaster Risk Reduction (ARSDRR), aiming to increase political commitment, enhance governance pertaining to DRR, and integrate DRR into emergency responses (African Union, 2004). The ARSDRR is implemented through regional economic blocs, a working group on DRR, and the respective national governments.

Kenya's DRR programme aligns with the Intergovernmental Authority on Development (IGAD)'s Disaster Risk Management programme, most notably, its Drought Disaster Resilience and Sustainability Initiative and its Regional Roadmap for Anticipatory Action, aimed at strengthening the resilience of vulnerable populations to climate shocks through early action and effective coordination (IGAD, 2019; see also Republic of Kenya, 2016b). The National Disaster Risk Management Policy, drafted in 2000 following the 1998 El Niño‐induced rainfall and the attack on the United States embassy in Nairobi, Kenya, was adopted in 2017 to strengthen institutional mechanisms for DRM and reduce disaster risk at both the county and national levels (Republic of Kenya, 2017). Beyond these policies that target all climate‐related hazards, such as floods, diseases, and fires, Kenya has specifically developed robust institutional policies governing drought management, which sometimes complicate how different agencies interact and mobilise resources to manage disasters. In what follows, I explore policy and institutional transformation in relation to drought management.

**FIGURE 2:**
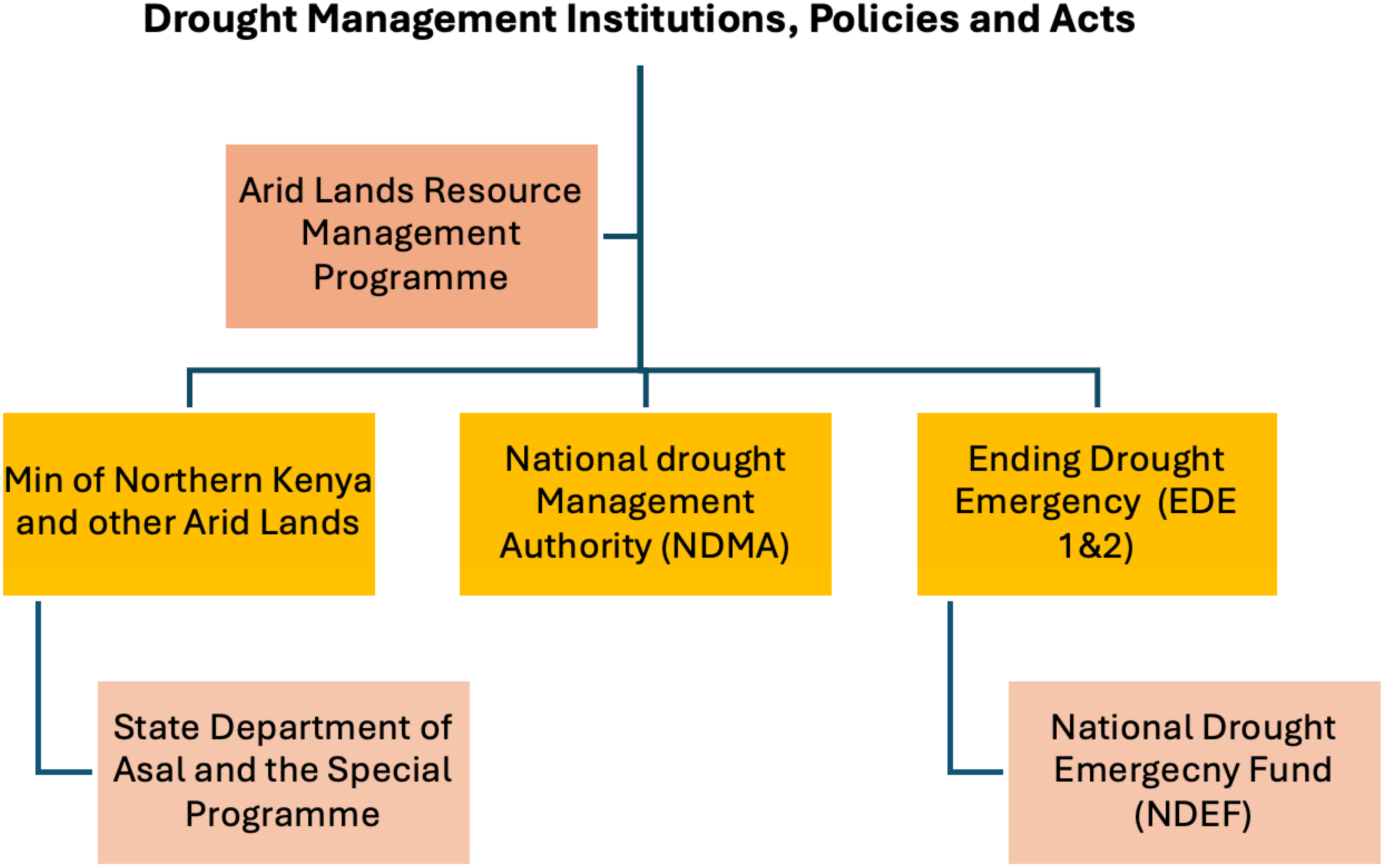
Selected drought management institutions and acts in Kenya. **Source:** author.

Kenya's institutional and legal frameworks for drought response have evolved substantially since the 1980s, beginning with the Arid Lands Resource Management Project and the Drought Management Initiatives (see Figure 2). Major policy shifts appertain to the conceptualisation of drought disasters and institutional changes—for an overview, see Derbyshire et al. (2024). The Ministry of Northern Kenya and Other Arid Lands (MNKOAL) was established in 2008 to address challenges in Kenya's arid and semi‐arid lands (ASALs), a region profoundly affected by prolonged drought. The MNKOAL was tasked with integrating the ASAL region into the national economy, ensuring climate resilience and promoting sustainable livelihoods (Odhiambo, 2014). This led to the establishment of the National Drought Management Authority (NDMA) in 2011, which coordinates drought management and acts as the secretariat for county steering groups (CSG) and the Ending Drought Emergencies (EDE) framework (Republic of Kenya, 2015). These entities and actors are bound by diverse organisational or sector‐specific strategic plans and mandates, which ultimately influence the uptake of policy goals.

The formation of drought risk management policies, institutions, and legal frameworks has yielded significant results, particularly in the areas of effective monitoring and early warning, which are key pillars of integrated drought management (Pischke and Stefanski, 2016). Early warning information systems have improved, including monthly bulletins and biannual food security assessment reports by the NDMA (2024). The Hunger Safety Net Programme (HSNP), a predictable cash transfer of KES 2,700 (USD 20) to vulnerable households across eight counties, is a notable programmatic shift that cushions the beneficiaries against chronic food insecurity. The HSNP lacks a beneficiary graduation model, though, thereby failing to improve overall resilience to shocks and recovery from persistent disasters (Rufo et al., 2025). It has, however, smoothened households' food consumption during crises (Sabates‐Wheeler et al., 2021).

The establishment of the National Drought Emergency Fund (NDEF), launched in 2012 under the Public Finance Management Act, and operationalised in 2021, is another milestone (Republic of Kenya, 2021). In fiscal year 2024, the Kenya National Treasury allocated KES 182 million to the strengthening of drought response and recovery through the NDEF, despite a promise to assign KES 2 billion annually (Republic of Kenya, 2024). The resource is designed to be redistributed across drought preparedness (50 per cent), response (40 per cent), recovery (5 per cent), and administration (5 per cent), but its operationalisation has been slow (NDMA, 2022). The NDEF could also overlap with the National Disaster Management Fund, established in 2022 (Republic of Kenya, 2022).

Despite these efforts and Kenya's explicit commitment to the global climate change and DRR agenda, significant gaps remain. Our findings established that shifting government regimes, a lack of political will to operationalise financial commitments, ministry realignments, and changing national priorities have constrained the effective operationalisation of the policies.

### 2.1 Conceptual framework

**FIGURE 3:**
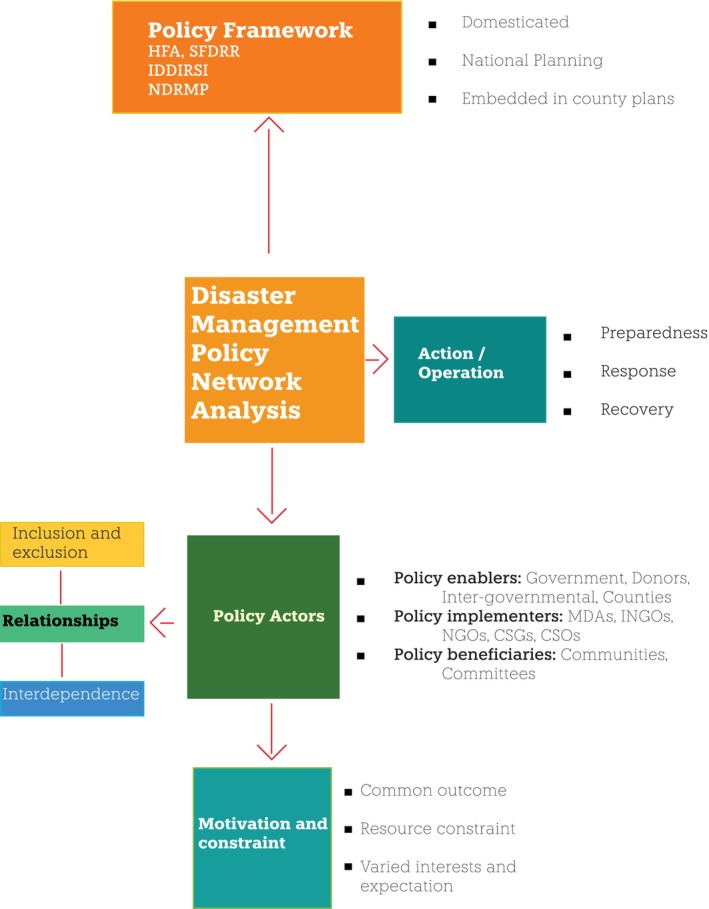
Diagram summarising the DRM policy network in Kenya. **Notes:** MDAs = ministries, departments, and agencies; INGO = international NGOs; CSGs = county steering groups; CSOs = civil society organisations. All other acronyms are spelt out in Figure 1. **Source:** author.

Drawing on policy network theory (Rhodes and Marsh, 1992; Schneider, 1992), this study takes relationships between policy actors as centred on interdependence, examining who is included and excluded, as well as the frequency of meetings and knowledge sharing. It also asks whether a relationship is mutual or conflicting. To assess the motivations and constraints for effective DRM, this investigation observed the forms of incentives, appraisal, power relations, conflicting political will, and resource constraints that characterise policy outcomes or contribute to the hidden dynamics that undermine policy implementation (Mosse and Lewis, 2005). These dynamics include static and often strict operational systems embedded in organisational structures, multiple perspectives, and actors' varied interests vis‐à‐vis policy discourse. Lastly, the analysis assessed actors' actions to integrate short‐term emergency response into long‐term development through the three phases of integrated disaster management (preparedness, response, and recovery). Figure 3 summarises this analysis.

## 3 METHODOLOGY

The study employed policy network analysis as a guiding tool to construct a broader ‘structure’ of DRM policies in the Greater Horn of Africa, with a focus on Kenya, and notably spotlighting drought management, a key national disaster (ADRA et al., 2021). It then mapped the ‘actors’ involved in operationalising DRM policies and the diverse ‘relationships’ between them. Lastly, it characterised incentives and constraints for coordinating these actors towards a collective outcome of a ‘disaster‐resilient’ community (Rhodes and Marsh, 1992; Schneider, 1992; Comfort and Haase, 2006; Kim and Hossain, 2013).

### 3.1 Research design

This study employed a phenomenological design to document the interactions among actors (that is, enablers, implementors, and beneficiaries) engaged in implementing disaster management policies in Kenya. Data were collected between May 2023 and September 2024 in three phases. The first of these entailed desk studies and stakeholder discussions, followed by a roundtable workshop to define the state of the field and to identify priority research areas. The second phase involved gathering empirical data from among international non‐governmental organisations (INGOs), development actors, donors, humanitarian organisations, and United Nations (UN) agencies based in Kenya, as well as those working in other countries, such as Ethiopia, Somalia, and Sudan. These institution‐based interviews provided a high level of understanding of overall disaster management policies in the Horn of Africa. They also highlighted how these diverse actors align their humanitarian and development programmes in relation to operationalising effective disaster management. The objective was to comprehend the scope of alignment of disaster preparedness, response, and recovery practices among international, national, and local actors and the degree to which they promote flexible operating systems.

Building on the institution‐based interviews, the third phase analysed the interactions between policy enablers, implementors, and beneficiaries from Marsabit and Isiolo Counties in northern Kenya. This provided a practical overview of how NGOs, CSOs, and both county and national government departments prepare for, respond to, and practice recovery post disaster. The participants were asked whether their DRM policies aligned with national, county, and community DRM plans. The study also examined how policy enablers, implementors, and beneficiaries interact, including by exploring the frequency of meetings, the sharing of information, and the relationships that exist between them. These lines of inquiry revealed a critical disconnect between DRM policies and local‐level implementation, with operations largely centred on response rather than early preparedness and recovery plans. Meanwhile, drawing together data on national‐ and international‐level policy formulation and local‐level implementation practices yielded a detailed understanding of how policies are conceived, interpreted, and utilised across different levels.

### 3.2 Study site

The empirical material for phase three of the research was amassed in northern Kenya's Marsabit and Isiolo Counties, both of which are arid areas regularly impacted by drought. The 2019 census recorded a population of 459,785 and 268,002 persons, respectively, in each of them (KNBS, 2019). Livelihoods pursued in Isiolo and Marsabit Counties are diverse, comprising a mixture of pastoralism, agro‐pastoralism, trade, casual work, and formal employment, often across single families. Both areas also experience frequent climate‐related disasters, conflict, food insecurity, and malnutrition, making them regular recipients of humanitarian and social assistance (Marshak et al., 2021; Mkutu, Müller‐Koné, and Owino, 2021; Mohamed and Scoones, 2022; Johnson et al., 2023; Mohamed and Scoones, 2023; Venkat et al., 2023).

### 3.3 Data collection and participants

The study combined empirical data collected over 18 months with secondary data sourced from scientific peer‐reviewed journals, evaluation reports, policy documents, and organisational websites. Notes from the secondary data were organised into three themes: humanitarian response to crisis; resilience programmes; and community‐managed DRM. Humanitarian and resilience programmes in the Horn of Africa between 2011 and 2024 were mapped to lay the foundation for an analysis of how humanitarian and development programmes and policies have evolved over a decade and how they manage disasters in the region. In May 2024, a workshop was organised for practitioners from financing agencies, the UN, humanitarian organisations, development NGOs, and the Kenyan government. The discussion focused on breaking down siloes among humanitarian and development actors (Mohamed et al., 2024). It emphasised the need for enhanced political will to translate policies into action, enabling effective coordination and service delivery—a debate out of which this paper emerged.

The study employed purposeful sampling, with participants identified through stakeholder dialogue, existing networks, and snowballing through referrals from interviewed respondents. The participants included humanitarian actors, development practitioners, researchers, government officials, disaster management committees, CSOs, UN agencies, INGOs, intergovernmental organisations, local NGOs, and disaster coordination bodies (see Table 1).

**TABLE 1:** Summary of interviews and methods used.

Name	Numbers conducted	Location	Total Participants	Agencies^1^
Key informant interviews	24	Nairobi, online	29	INGOs:11
Stakeholder dialogues	11	Nairobi, online	23	NGOs^2^: 11
Focus‐group discussions	4	Isiolo, Marsabit	24	UN^3^ agencies: 4
Roundtable workshop	1	Nairobi	33	Bank^4^: 1
**Total**	**40**		**109**	Government^5^: 8 Donors^6^: 4 Community: 8 Intergovernmental organisation^7^: 3

**Source:** author.

^1^ INGOs: Action Against Hunger, ADRA Africa, International Federation of Red Cross and Red Crescent Societies, International Livestock Research Institute, Kenya Red Cross Society, Mercy Corps, Oxfam, Relief International, Save the Children, Welthungerhilfe, and World Vision.

^2^ ASAL Humanitarian Network (AHN), Busara, Centre for Humanitarian Change (CHC), Centre for Research and Development in Drylands (CRDD), Drylands Learning and Capacity Building Initiative (DLCI), Indigenous Movement for Peace Advancement and Conflict Transformation (IMPACT), Marsabit Women Advocacy and Development Organisation (MAWADO), Merti Integrated Development Programme (MID‐P), Pastoralist Community Initiative and Development Assistance (PACIDA), Pastoralists Alliance for Resilience and Adaptation Across Nations (PARAAN), and Reconcile.

^3^ Food and Agriculture Organisation of the United Nations (FAO), United Nations Development Programme (UNDP), United Nations Office for the Coordination of Humanitarian Affairs (UN OCHA), and the World Food Programme (WFP).

^4^ African Development Bank.

^5^ County officials, national government officials, the National Drought Management Authority (NDMA), and the National Disaster Operations Centre (NDOC).

^6^ International Development Research Centre (IDRC), Foreign, Commonwealth & Development Office (FCDO), Swiss Agency for Development and Cooperation (SDC), and the United States Agency for International Development (USAID).

^7^ IGAD Intergovernmental Authority on Development, IGAD Climate Prediction and Application Centre (ICPAC), IGAD Centre for Pastoral Areas and Livestock Development (ICPALD).

## 4 DISASTER MANAGEMENT INSTITUTIONS: THE CASE OF MARSABIT AND ISIOLO COUNTIES

**FIGURE 4:**
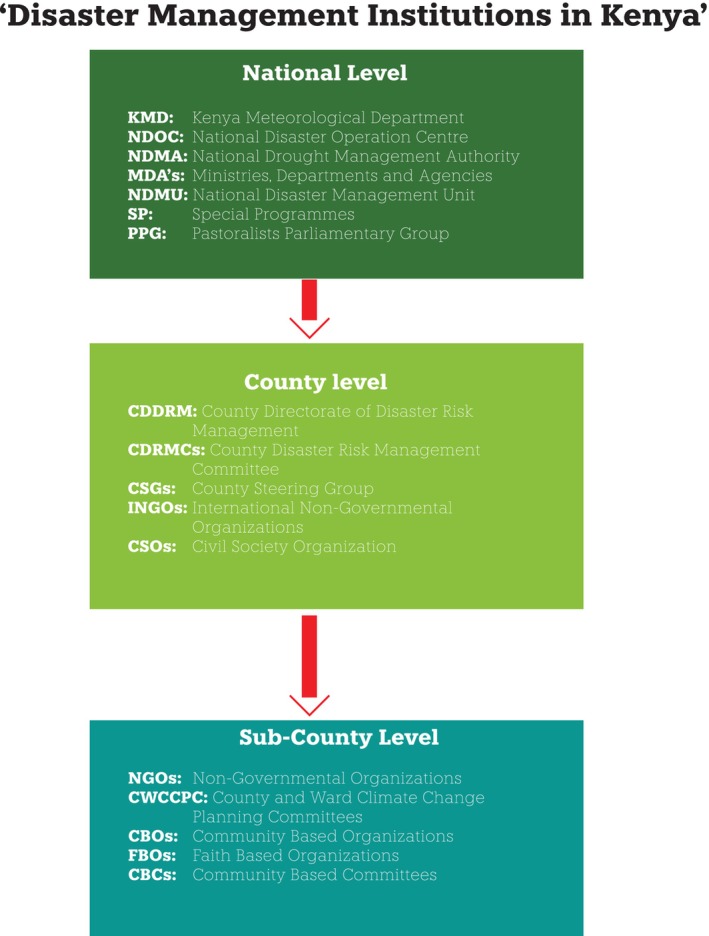
Diagram summarising the structure of disaster management institutions in Kenya. **Source:** author.

Various institutions and policies have contributed to disaster preparedness, response, and recovery in Kenya's arid and semi‐arid counties. These include a caucus in parliament called the Pastoralist Parliamentary Group (PPG), which convenes regularly with the aim of mainstreaming challenges faced by disaster‐prone pastoralist counties in national planning and resource allocation. CSGs, comprising county government departments, national government agencies, and CSOs, coordinate disaster response and recovery efforts. Ultimately, community groups and committees facilitate connections between disaster‐affected communities and the broader world of disaster response stakeholders. This section provides an overview of these institutions and examines the relationships among the actors within them. It analyses the factors influencing policy outcomes for disaster‐resilient communities and how interactions between actors enable or constrain such endeavours Figure 4.

In terms of institutions, the Kenya Meteorological Department provides climatological and meteorological forecasts to various sectors involved in DRM. Different government ministries, departments, and agencies (MDAs), such as the National Climate Change Secretariat, housed within the Ministry of Environment and Natural Resources, coordinates the activities of government ministries and departments related to CCA (Republic of Kenya, 2016a). It supports the Ministry of Devolution and Planning in mainstreaming climate change in Medium‐Term Plans and County Integrated Development Plans. Within the Ministry of Devolution and Planning, the State Department for Special Programmes leads disaster response and relief provision. However, the National Disaster Operation Centre (NDOC), established in 1998 as a department under the then Ministry of Internal Security, coordinates, monitors, and mobilises resources to respond to disasters at the national level (Republic of Kenya, 2017).

The National Disaster Management Unit (NDMU), established by a presidential directive in 2013 as an inter‐agency unit led by the National Police Service, coordinates disaster preparedness, response, and recovery (NDMU, 2020). The NDMU has a command structure and standard operating procedures that are organised across national operation structures and county disaster operations. The NDMA, meanwhile, coordinates drought response and recovery in the country.

The NDMA, NDOC, and NDMU link the national disaster policy to county‐specific disaster response plans. In Marsabit and Isiolo Counties, the NDMA coordinates disaster response (although it is broadly a drought management institution). Multiple interview responses highlighted limited coordination among these three national institutions, as each was formed for different disasters and managed by unrelated institutions and ministries, with unconsolidated budgetary allocations. There has been an attempt to bring all of these agencies under one umbrella, through the National Disaster Risk Management Bill (Republic of Kenya, 2023), but the legislation has stalled in parliament. Indeed, a respondent from the Marsabit county government stated that ‘disaster coordination is unstructured, and the motivation and finance to effect coordination are not there. In fact, coordination is mostly pegged to funding, not issues. NDOC and NDMU are not involved in CSGs, and since NDMA deals with drought, we struggle with how to finance other compounding disasters’.

The PPG has been vocal in mainstreaming the pastoralist agenda in national planning. Established in 1990, the Group is primarily concerned with the legislative branch of policy. It convenes through the Pastoralists' Leadership Summit (PLS), a three‐day gathering of political leaders who deliberate issues that affect pastoral areas. The PLS lobbies for strategic demands from the national government, including recent financing of EDE 1 and 2. Resolutions from the PLS Summit 2019 in Garissa County called for the immediate disbursement of equalisation funds (to alleviate the legacies of historical marginalisation in arid and semi‐arid counties) and increased budgetary allocation for the livestock sector (to a minimum of 10 per cent) (PPG, 2019). PLS Summit 2024 facilitated the establishment of a livestock development and promotion service (Kitel, 2025). The PLS leadership has also facilitated infrastructure development in northern Kenya and the institutionalisation of key bodies, including the State Department for Special Programmes and the NDMA (PPG, 2016).

In this regard, PPG lobbying has, broadly, improved overall disaster response in Kenya's pastoral counties; however, it faces slow progress in affecting change owing to competing political and national agendas. A conversation with a prominent leader at the PLS Summit 2024 revealed that ‘although PPG is the largest caucus in the National Assembly, the lobbying for a change in policy and advocacy for drylands development is slow. It often faces backlash from the political class, who feel threatened by the preferential treatment of the northern pastoral counties. They don't understand that these counties were left behind because of political–economic marginalisation, and it is time to rectify those mistakes’.

CSGs comprise county government executive committees, national government departments, INGOs, NGOs, CSOs, and the private sector. The county governor and commissioner co‐chair these bodies, with the NDMA serving as the secretariat. CSGs hold quarterly stakeholder meetings; the frequency is increased during active disasters, including drought emergencies. The County Directorate of Disaster Risk Management oversees the disaster response and is represented at CSG meetings. CSG members indicated that these steering groups provide a message conveyance space but lack the capacity and finance for post‐disaster recovery. A CSO informant in Marsabit said: ‘CSGs are an excellent forum for sharing information and overseeing activities, ensuring no duplication; however, they are always caught unawares and unprepared when disasters occur. They are under‐resourced and continuously seek even minor support’. Other interviewees repeated the same sentiments.

CSOs in Isiolo and Marsabit Counties work under the ASAL Humanitarian Network (AHN), which was established in 2019. The AHN operates in all of the arid and semi‐arid counties to enhance local disaster response and adaptation. As a platform, it enables members to promote the localisation of humanitarian action and capacity development among their peers. A member of the AHN in Marsabit described the network as follows:

*AHN is a network of 30 NGOs from the ASAL counties. We convene quarterly meetings and increase the frequency during times of crisis. We use NDMA, TV, and radio alerts to prepare for disaster response. As CSOs, we independently source funds to complement those of the national and county governments, serving the same community. We prioritise areas needing support through the AHN Steering Committee, chaired by the Secretariat. During the 2022 drought, we identified Garissa and Tana River Counties as the most affected, and we diverted resources through our network to these two counties*.

At the community level, disaster response service providers, including CSOs, county governments, and government departments have also established various committees. The most common approaches in Marsabit and Isiolo are survivor–community‐led response (SCLR) and community‐managed disaster risk reduction (CMDRR). CMDRR and SCLR are practices where disaster‐affected communities receive resources to manage disasters by planning, preventing, and responding to crises (Cordaid, 2013; AHN, 2023). This is achieved by ‘empowering the community’, instituting local management committees and strengthening mutual learning and risk assessment. Current disaster response programmes in both counties have attempted to embed CMDRR and SCLR principles by establishing multiple project committees. This process aims to centre the affected community as the first and last responders to crises based on existing norms, knowledge, and resources. Yet, as I discuss in the subsequent section, such community committee formations are often undermined by community power dynamics and there are limited resources to sustain the structure.

In Marsabit and Isiolo, these committees include the Participatory Disaster Risk Assessment Committee, the County and Ward Climate Change Planning Committee (WCCPC), ‘community champions’ (individuals who communicate early warning information from the NDMA), and the rangelands committee supported by conservancy‐promoting agencies such as the Northern Rangelands Trust. Equally, sectoral‐based committees representing health (Community Health Promoters), land, water, grazing, nutrition, and other sectors are prominent. Some of them play a crucial role in bridging the gap between communities and service providers, developing contingency scenario plans, reporting disaster outbreaks, and communicating with their communities on disaster prevention and preparedness. However, study participants noted that numerous committees have become a mechanism for project ‘box ticking’ under the guise of participatory community‐led interventions. Many become non‐operational after project inception meetings. Study participants observed a distinct lack of a mechanism for sustaining and evaluating the performance of existing committees. Instead, there was rampant formation of new committees. A member of the WCCPC in Isiolo explained:

*The community elects the WCCPC to represent them in ward‐planning activities. We usually draft ward development plans, including contingency and community disaster risk reduction plans. During disasters, such as floods or disease outbreaks, we inform ward administrators, who then connect us with the relevant county government officials. Our challenge is that when the county implements activities from ward development plans, it forms a new project committee, sidelining the previous committee. The community elects us, but the county government claims we are its employees and can only speak on its behalf, not on behalf of the community. They threaten us during meetings and say they will ban the committee if we don't act accordingly*.

Thus far, this section has highlighted the existing institutions in place for disaster management, ranging from high‐level policy‐enabling parliamentary groups and county government‐led steering groups to civil society and community‐level committees. The next section explores some of the ways in which the relationships and interactions among actors in these institutions influence how disasters are managed.

### 4.1 Relationships, networks, and motivations among DRR actors

Table 2 summarises the analytical components characterising the relationships and interactions among actors in the disaster management cycle. Interactions between DRM policy enablers (primarily donors and national governments) take place through a distant relationship facilitated by bilateral arrangements with recipient governments. They also occur through contracting INGOs, UN agencies, local NGOs, and government departments in the recipient country. Meetings between these actors are infrequent and usually involve exchanging quarterly or annual reports to assess ongoing humanitarian or development interventions. These agencies subcontract local NGOs and CSOs that interact directly with beneficiaries. Contracting and subcontracting through these diverse actors is time‐consuming, and given the short‐term nature of DRM programmes, there is a tendency to rush interventions to enhance the ‘burn rate’ without due consideration of post‐disaster recovery. Consequently, disaster management overlooks the significance of an ‘integrated’ approach that combines preparedness, response, and recovery.

**TABLE 2:** Components and variables of policy network analysis.

Components	Variable	Description
Participants	Actors	Enablers, implementors, and beneficiaries
Relationship	Frequency of meetings	Interactions or no meetings?
	Direction	One‐sided or mutual?
Operation	Preparedness	Cooperative or conflictive
	Response	Reactive or proactive
	Recovery	Inclusive or exclusive

**Source:** author, adapted from Kim and Hossain (2013).

At the CSG level, enablers and implementors meet quarterly, but the frequency increases to once or twice a month during droughts and other emergencies. These meetings involve the sharing of disaster information and assess partners' interventions to avoid duplication; but there is no integrated plan for the disaster preparedness, response, and recovery phases. A CSG member from a government agency pointed out that the ‘CSG satisfies donors and community interests, mapping vulnerable areas and needs. However, it fails to evaluate intervention outcomes, such as the lives saved from malnutrition or the effectiveness of post‐disaster recovery plans. It operates as an agency‐approving action without a collective evaluation of outcomes’. Equally, within CSG meetings, beyond the partner exchange forum, there are limited interactions pertaining to the division of labour and resources at each disaster management stage (preparedness, response, and recovery). As noted, the frequency of meetings rises at the peak of a disaster, focusing on those with immediate relief resources. The diversity of actors fulfils immediate response needs but lacks complementarity for sustained recovery and resilience.

One key observation from the study is that at a higher level, relationships are particularly one‐sided (that is, each agency claims to lead disaster responses). This means that there is a lack of significant coordination of efforts in reconciling resources to respond to slow‐onset events, such as droughts. A county government respondent stressed: ‘We are supposed to work with NDOC, NDMU, and other MDAs but they are based in Nairobi, and all are not represented at the CSG, although CSG provides a crucial platform for governing how disasters are managed. Sadly, northern Kenya's disasters are mostly left with the NDMA. And yet, aside from drought, we suffer from other complex disasters such as floods, locust swarms, and animal diseases, which require a multi‐agency response and recovery plan under good governance and coordination’.

Community volunteers or committees serve as the last element of disaster management. These groups are often trained in DRM and play a crucial role in linking the community to external stakeholders. While most volunteers are trusted by their communities and frequently assumed to enhance ‘participatory’ approaches, some tension exists, especially when benefits such as allowances and remuneration for workshop attendance are involved—a dynamic also observed by Watkins and Swidler (2013) in Malawi. These authors noted that the conflicting interests and worldviews of aid actors often lead to a particular way of ‘working’, maintaining the status quo and leaving little room for learning and improvisation. In the same way, the disaster management committees' roles are often predefined and tied to standardised ‘community engagement’, which is done excessively by all implementing agencies. These committees disseminate information through local forums, but how beneficiaries receive and act on it is often not assessed, leaving the process one‐sided.

For community members, responding to a disaster is a continuous process that involves relying on previous knowledge, practices, and multiple social and economic relationships. A herd owner from Isiolo commented: ‘When a disaster such as a drought hits, we diversify activities, [determine which] stock requires medicine for the dry season, negotiate access to restricted areas such as national parks and private farms for pasture, and scout for safe places for grazing. We cannot do all of these unless we establish trust and good networks with our neighbours, agrovet shops, traditional vets, and motorcycle riders for easy transportation’. In short, an array of networks, social relations, local assistance, knowledge, technologies, and reliable sources of finance enables disaster preparedness, response, and recovery on a rolling basis.

The relationships among enablers, implementors, and beneficiaries are characterised by complex expectations, varied experiences and interests, and different aspirations, which are difficult to reconcile. These actors find common ground in ‘coordination’ meetings. They use ‘coordination’ as leverage to make disaster response effective, but none are willing to change their operations to fit wider systemic needs. Fundamental ideas about disaster preparedness, response, and recovery differ among entities, and reconciling these worldviews is challenging. A key interview respondent said: ‘When the resilience agenda emerged, humanitarian actors were tasked with combining development components into their emergency intervention. As much as this appears doable, the actor's inherent mindset cannot be changed to accommodate the new order’. Actors find little space for learning and effecting disaster response changes on a network‐wide level. This is primarily because of constraints caused by institutional and policy factors, which I explore in the following subsection.

### 4.2 Constraints and motivations for DRM

Policy directives are steps towards achieving intended goals but require strong institutions, governance, resources, and motivation. Operationalising policy is a slow and often lengthy process, potentially taking decades. For example, the NDEF was ‘launched’ in 2012 but was not instituted until 2021. This study found that organisational mindsets, diverse political agendas, resource constraints, and complex institutional interests are key factors contributing to the slow adoption of policies. Overlapping mandates between organisations and transition between institutions and stakeholders in the policymaking arena further contribute to the ineffective operationalisation of policies. For instance, the Kenya National Treasury (2024) has developed public disaster management funds, which are regulated under the Public Finance Management Regulations, 2022, to facilitate disaster preparedness, response, and recovery. Although these funds are intended to consolidate all disaster‐related funds, they may potentially overlap with other existing disaster funds, such as the NDEF.

Diverse institutional mandates and mindsets bind DRM stakeholders. A key informant from a humanitarian agency remarked: ‘The mandate itself is an inhibitor; we are naturally—and I think sometimes a little problematically—short term in our thinking. Even development actors suffer from a short‐term programme cycle of two to three years. The first year is spent on planning and assessing community needs, which is more valuable; however, operational managers are evaluated on delivery rather than quality. We then prioritise what is written on the programming paper and mindlessly do things, sometimes, unfortunately, with legacy minds and self‐interest’. Such sentiments emerged repeatedly throughout the study, highlighting how actors struggle to separate operational practices from organisational culture, prioritising aid delivery over effectiveness.

Beyond this recurring theme of skewed priorities, many study participants also highlighted a broad lack of trust in data or the link between data and action (and, in this sense, a lack of confidence in the value of data). A participant from one prominent INGO stressed: ‘When I was a donor, I never believed the report was 100 per cent positive. I never read a report with a trusting attitude: documents that said 95 per cent of the community loved what we did were rubbish. This is what these guys thought we needed to hear’. In a different interview, an NGO worker added: ‘A lack of evidence does not limit us, but a lack of trust in the data [does]. We had 42 indicators for the 2022 droughts, and the drought warnings were flashing, but the investment came late. This is not the system's problem; it is operationalising the system’.

Often, as part of these conversations around data, accountability also emerged as a key theme, particularly the lack of mechanisms for assessing progress and bottlenecks in disaster preparedness and recovery. A prevailing sentiment was that strong institutional leadership—something drastically required—is absent during the disaster response process. Indeed, a key informant stated: ‘We cannot decide how an intervention is done; we just accept it; after all, it is an aid’. Accountability entails ownership of maladaptation and the capacity to ask the right questions for improvement. Efforts have emerged through Transparency International and its partners, who have established *Uwajibakaji Pamoja* (accountability together), an integrated complaint referral system in four arid and semi‐arid counties to improve the capacity of service providers and beneficiaries for quality aid interventions (Transparency International Kenya, 2023). Launched in Marsabit County in 2017, it was not operational as of 2025 and the county government, perhaps somewhat unsurprisingly, lacked resources for its continuation.

Negligible incentives exist for aligning humanitarian aid with developmental interventions or embedding disaster preparedness, response, and recovery in programming. Instead, disaster risk actors are more oriented towards immediate disaster response and less so with respect to long‐term development and recovery. One study participant from an INGO pointed out: ‘There was not enough reason to change operations without incentives. We lack such culture and are imbued with structures, frameworks, and plans’. In a similar vein, a respondent from an NGO in Isiolo said: ‘moral values and honesty should guide us to deliver more than strategy and frameworks for ending disasters. Unfortunately, emergencies are often seen as opportunities for theft because of reduced auditing’. Additionally, study participants, both during the workshop and among key informants, noted that key performance indicators, combined with more flexible financing, could incentivise DRM actors to align their activities for collective outcomes; however, such efforts require strong leadership from the government at all levels of disaster management.

Lastly, many interviewees highlighted concerns about limited financial and human resources, widely perceived as undermining effective coordination of disaster response and recovery. It is worth emphasising that there is no funding earmarked for integrating development and humanitarian activities beyond crisis modifiers and contingency funds, which are frequently either neglected or bureaucratically extremely difficult to operationalise. A respondent from an intergovernmental organisation noted: ‘We have robust DRM tools and coordinating bodies, but they are often designed for resource mobilisation. When funds dry up, they lose focus and prioritise resource‐seeking for survival’. All of the stakeholders interviewed affirmed that resource constraints, especially inflexible and input‐focused financing, were an inhibiting factor in operationalising disaster management policies. This is undoubtedly exacerbated by Kenya's growing debt crisis, which hinders access to concessional loans and grants, forcing the government to reallocate development funding for disaster response, instead of investing in preparedness and recovery.

## 5 NAVIGATING CHALLENGES AND OPPORTUNITIES IN DISASTER MANAGEMENT: LESSONS FROM POLICY NETWORK ANALYSIS

Studying complex institutional relationships, expectations, and interests in disaster management systems to determine key opportunities for improvement in response and recovery is fundamental to making progress. As Cooley and Ron (2002) set out, the power dynamics and complex operations within government agencies and their partners in relation to disaster management create an environment and objectives that either enable or hinder outcomes. If this environment is to be improved, it needs to be carefully understood. A key insight from policy network analysis is that navigating complex disaster management policies requires strong bonding relations based on trust, good governance, accountability, and collective solidarity, rooted in human relationships.

A helpful idea here is Andrew and Carr's (2013) discussion of ‘bonding’ relationships, mentioned earlier, which play an active role within single agencies and are driven by ‘thick trust’, and ‘bridging relationships’, which form between actors from different agencies and are often characterised by ‘thin trust’. Putnam's (2000) original conceptualisation of these various relationships suggested that bonding relationships are about ‘getting by’, while bridging relationships are about ‘getting ahead’. Bringing these ideas into conversation with policy network and institutional perspectives, the study found that agencies such as CSGs, which convene all actors engaged in DRM at the county level, emulate a bridging relationship that facilitates preparedness, response, and recovery but is not utilised accordingly. CSGs should capitalise on their convening power and bridging capacity to encourage trust, accountability, and good governance among the actors involved in DRM.

Multiple disaster management institutions in Kenya superficially emulate ‘bridging relationships’ with thin trust but work to stifle and suppress them via underlying dynamics. CSOs such as the AHN, for example, are established to coordinate disaster preparedness across multiple actors, a critical collaborative role that is hindered by standardised operating practices and cultures within each agency. How might new, more robust bridging relationships form across these groups, in the face of limiting contextual factors? One possibility is for cultural shifts led by senior management (Levine et al., 2020) to take place among policy implementors, enablers, and beneficiaries. More flexible working environments that allow actors to improvise, learn from mistakes and successes, and improve overall operations may need more decisive, future‐focused leadership that is primarily oriented towards wider systems, networks, and challenges. A key question in considering this is: what incentives might facilitate such leadership? Important insights can be drawn from pastoralist communities themselves, which navigate complex crises through local knowledge, mutual assistance, and deeply entrenched durable bonding relationships (cf. Caravani et al., 2022; Mohamed, 2023).

Collaborating amidst profound uncertainty, pastoralists draw on past experience to improvise and respond dynamically to the shifting terrain of challenges and possibilities. A strong underlying sense of solidarity, built around bonds of mutual obligation and shared responsibility, is what makes this possible. Is such an impetus conceivable in high‐level disaster management planning? Perhaps not in any simplistic sense. It is an important model to reflect on, nonetheless, if only in its stark divergence from the modus operandi shaping disaster management across multiple levels. It demonstrates what can happen through more committed forms of collaboration, even with minimal access to resources and restrictive working conditions in the context of global aid cuts.

Competition and conflicting worldviews negatively shape efforts to reduce vulnerability to climate risk, creating winners and losers in situations that should be collaborative. Watkins and Swidler (2013) show this in a study on how fraught relationships between HIV/AIDS (Human Immunodeficiency Virus/Acquired Immunodeficiency Syndrome) actors in rural Malawi create misunderstanding and diverse aspirations. Similarly, in her study of Vietnam's capital‐intensive adaptation programme, Thomas (2023) flagged how the plethora of adaptation interventions perpetuate competition while actors haphazardly configure practices that work for ‘everyone’ without questioning the value and impact of the interventions themselves. Likewise, disaster management actors in Kenya collaborate only to the extent that their respective responses are acceptable to all agencies involved, without being critical of malpractice or failure, and in this sense, without a view to improving approaches to the core issues at stake. A key example of this is the way that CSGs convene DRM actors without any scope for governance or strict accountability measures. Instead, each agency is allocated a territory. This allocation depends on its previous investment paths rather than on the effectiveness of its work.

Overall, this study suggests that new ways of working and ultimately improving disaster management will not come from new frameworks, policies, and strategies, but rather from introspection and new approaches to collaboration and knowledge management. This message is particularly crucial in an era increasingly characterised by shrinking aid budgets and global economic uncertainty. Doing more with less requires solidarity, cohesion, and a shared vision, rooted in bonding and bridging relationships. Nurturing these is extremely difficult amidst forms of ‘upward accountability’ that relentlessly pull the focus away from core issues, places, and peoples (Mohamed et al., 2025). One way of addressing this might be the development of performance metrics that better reward inter‐agency cooperation, accountability to affected communities, transparent reporting systems (including when interventions fail to meet desired goals), and intentional resource allocation during national budgeting and planning. This is perhaps only possible via leadership that looks beyond new frameworks and towards longer‐term, broader‐scale objectives.

## 6 CONCLUSION

Through an assessment of actor relationships and interactions across various levels of disaster management, this study has identified critical disconnects between disaster management policies and institutions, as well as the actors responsible for implementing the policies. These disconnects emerge from hidden dynamics and are only discernible via careful, network‐wide examination of institutional relationships and practices. In addressing these disconnects, there is a crucial need to capitalise on the kinds of ‘bonding’ relationships that foster trust and accountability. Ultimately, this is the only way to instil a broader sense of confidence in the relationship between data and action. Regarding the specific case study examined in this paper, Kenya, CSGs appear to be a critical context for the development of new, more collaborative approaches. Their role is both deeply significant to wider ideals of improved collaboration, openness, and collegiality, and yet is drastically hampered by dynamics that undermine collective interests. Instead, CSGs are entrenched in a ‘bridging’ relationship, providing a platform for collaboration, but they lack the capacity and authority to monitor, evaluate, and influence the outcomes of disasters.

Overcoming financial, technological, and human resource constraints in disaster management requires that we rethink some of the fundamentals; there can never be sufficient resources to manage crises. In this regard, capitalising on existing disaster management finance, such as the NDEF, and instituting ways to enhance its operation are crucial. Such efforts include advocacy by the PPG during national planning and budgeting to prioritise the NDEF while also encouraging disaster‐affected counties to allocate advanced resources and invest in community DRM capacities. Equally vital is investing in local resources that support first responses rather than untimely aid (Scoones, Ling, and Allouche, 2024). Such local responses include flexible mobility, redistributive solidarities such as *zakat* (a wealth tax), livestock transfers, and mutual informal social assistance, which people rely on to avert disasters (cf. Mohamed, 2022). If carefully supported, such collective practices can help disaster‐affected communities overcome crises, even in fragile, conflict‐affected regions (Caravani et al., 2022).

Future research and policy initiatives in the field of disaster management should prioritise the addressing of these challenges, with a particular focus on finding solutions to the protracted nature of policy implementation. Consideration should be given to methods of incentivising governmental and other policy‐enabling institutions to reinforce existing institutional and systemic relationships rather than create redundant disaster management tools. Ultimately, approaches to developing more robust and streamlined disaster response must be linked to and oriented towards existing dynamics of institutional actors and evolving policies. Achieving this requires concerted efforts by all policy actors, including government entities, international partners, and local communities. Alongside this, improvements must be made in institutional relationships themselves to build trust proactively. Regular, candid dialogue is the key ingredient in this respect and is the only means of fostering collaboration towards the shared objective of creating a disaster‐resilient society.

## CONFLICT OF INTEREST STATEMENT

The author declares no conflict of interest in the authoring and publishing of this paper.

## ETHICS STATEMENT

This research was approved by the International Livestock Research Institute's ethical review board.

## Data Availability

The data that support the findings of this study are available on request from the corresponding author. The data are not publicly available due to privacy or ethical restrictions.
